# Baseline participation in a health examination survey of the population 65 years and older: who is missed and why?

**DOI:** 10.1186/s12877-016-0185-6

**Published:** 2016-01-19

**Authors:** Beate Gaertner, Ina Seitz, Judith Fuchs, Markus A. Busch, Martin Holzhausen, Peter Martus, Christa Scheidt-Nave

**Affiliations:** Department of Epidemiology and Health Monitoring, Robert-Koch-Institute, General-Pape-Str. 62-66, D-12101 Berlin, Germany; Institute of Biometry and Clinical Epidemiology, Charité - University Medicine Berlin, Hindenburgdamm 30, D-12203 Berlin, Germany; Department of Clinicial Epidemiology and Applied Biometry, Eberhard Karls Universität, Silcherstr. 5, D-72076 Tübingen, Germany

**Keywords:** Aging population, Non-participation, Public health monitoring, Reasons for non-participation, Register-based population, Selection bias

## Abstract

**Background:**

Public health monitoring depends on valid health and disability estimates in the population 65+ years. This is hampered by high non-participation rates in this age group. There is limited insight into size and direction of potential baseline selection bias.

**Methods:**

We analyzed baseline non-participation in a register-based random sample of 1481 inner-city residents 65+ years, invited to a health examination survey according to demographics available for the entire sample, self-report information as available and reasons for non-participation. One year after recruitment, non-responders were revisited to assess their reasons.

**Results:**

Five groups defined by participation status were differentiated: participants (*N* = 299), persons who had died or moved (*N* = 173), those who declined participation, but answered a short questionnaire (*N* = 384), those who declined participation and the short questionnaire (*N* = 324), and non-responders (*N* = 301). The results confirm substantial baseline selection bias with significant underrepresentation of persons 85+ years, persons in residential care or from disadvantaged neighborhoods, with lower education, foreign citizenship, or lower health-related quality of life. Finally, reasons for non-participation could be identified for 78 % of all non-participants, including 183 non-responders.

**Conclusion:**

A diversity in health problems and barriers to participation exists among non-participants. Innovative study designs are needed for public health monitoring in aging populations.

## Background

In recent decades, population-based health surveys have been facing decreasing participation rates [1]. Especially health studies of the older population are plagued by high non-response rates [e.g., 2–4]. This could compromise the validity of study results. At the same time valid estimates of health status, health risks and health care needs in the population 65+ years are urgently needed due to population aging.

Studies of non-participation conducted up to the 1990ies have often not included or sufficiently reported results for individuals 65+ years [e.g., 5–7], or left out the oldest old, i.e. those 80/85+ years [e.g., 8, 9]. The number of studies dealing with non-participation in health studies of older adults has increased since the 1990ies [2–4, 10–27], although many of these studies have focused on varying subsets of older persons with specific health problems, e.g. falls [4, 13], respiratory health [15], visual impairment [21] or rheumatoid arthritis [24].

The majority of health studies of older persons that investigated non-participation found non-participants are less well educated, have lower income, and live more often in residential care than participants [2–4, 12, 14, 15, 21, 25, 27]. However, regarding sex, marital status and subjective health status, findings have been inconsistent. For example, married individuals were found to participate less often than [17, 26], more often than [3, 4, 16, 25, 26] and to the same extent than non-married individuals [18, 20, 22, 26, 27]. Immigration background was rarely considered; one Dutch study reported no difference in response rates according to first language [18].

Although qualitative studies have generated lists of potential reasons for non-participation in intervention studies among older individuals, the frequency of single reasons could not be quantified [13, 23]. Reasons for non-participation of the older population appear to have changed over time. Studies from the 1960ies and 1970ies noted negative opinions about the health care system and health research studies in general [11, 25]; since the 1990ies, ill health and lacking time or interest have been the predominant reasons for non-participation [4, 8, 10, 12, 16, 19, 20, 26].

The aims of our study were twofold: (1) to assess baseline differences between participants and non-participants in a population register-based health examination study of adults 65+ years (including the oldest old), and (2) to analyze reasons for non-participation at baseline.

## Methods

The sample is part of the research project ‘Operationalizing Multimorbidity and Autonomy for Health Services Research in Aging Populations’ (OMAHA). The project was conducted as part of the German collaborative research initiative on health in older populations supported by the Federal Ministry of Research and Education (Germany). The project was approved by the local ethics committee at Charité – Universitätsmedizin Berlin (EA2/066/08) and was conducted in compliance with data protection and privacy regulations, as requested by the Federal and Berlin Offices for the Protection of Data. All procedures performed were in accordance with the 1964 Helsinki declaration and its later amendments or comparable ethical standards. Informed written consent was obtained from all participants.

### Sampling frame and participants

As described elsewhere in detail [28], OMAHA was conducted as a population-based longitudinal epidemiological study of multimorbidity and associated health care needs in an urban population aged 65+ years between January 2009 and January 2011. A random sample (*N* = 2000), stratified by age bands (65–69, 70–74, 75–79, 80–84, 85+ years) and sex was drawn from the official register of residents in Berlin-Mitte on July 15, 2008, including a total of 1481 persons for the main project and 519 for a pilot project. The stratified sampling procedure resulted in *n* = 200 individuals per age band and sex category. The drawing probability was higher in older age groups (especially of those 80+ years). Therefore, older individuals were oversampled. Inclusion criteria were permanent residence in Berlin-Mitte and being 65+ years. Individuals who had died, had moved outside of the study area or were continuously absent during the recruitment period were excluded from the study and considered *ineligible.*

Individuals were initially contacted by postal mail including a form to request a brief study description in seven different languages to address major immigrant subgroups in Berlin (Arabic, Croatian, English, Polish, Russian, Serbian, Turkish). Participants had the choice of home visits or appointments at the inner-city study center. A small monetary incentive (€ 10) plus reimbursement for travel expenses were offered. Individuals who did not respond to this invitation were further contacted randomly by personal visits, telephone calls, or reminder letters.

Baseline recruitment and assessment were conducted between January and June 2009 by trained and continuously supervised study nurses and a study physician. Study procedures included a comprehensive standardized computer-assisted personal interview (CAPI; e.g. medical history, instrumental activities of daily living), standardized functional capacity tests and physiological measurements (e. g. grip strength, blood pressure), detailed assessment of currently used medications, and a self-administered questionnaire (e.g. health-related behaviors, health care utilization).

Individuals who declined baseline participation were asked to answer a short standardized health questionnaire as a self-filled mail survey questionnaire (available in various languages) or via telephone interview. Proxy responses were allowed.

Three mutually exclusive groups of baseline non-participation according to their reachability were differentiated: (a) non-participants *with* the short questionnaire (NP+), i.e., individuals who declined study participation but completed the short questionnaire; (b) non-participants *without* the short questionnaire (NP-), i.e., individuals who declined study participation as well as the completion of the questionnaire; and (c) *non-*respondents (NR), i.e., individuals who could not be reached during the recruitment period and who did not actively decline study participation.

To further characterize non-participants at baseline, we assessed reasons for non-participation at two points in time. First, during the recruitment period, multiple reasons for non-participation could be specified by either self-report or proxy–reporting through postal, telephone or personal contact. Second, between July and September 2010, NR were revisited by a study nurse to retrospectively identify their reasons for non-participation at baseline.

### Measures

#### Register-based information

*Demographics* (age, sex, citizenship) and postal addresses were provided by the official resident register for the total sample. *Citizenship* was categorized into German vs. non-German. Postal addresses were checked by internet research for registered *residential care* (yes/no). Postal addresses were considered as being in a *deprived neighborhood* (yes/no) if the proportion of long-term unemployed (i.e., ≥one year) in the neighborhood was in the highest septile of Berlin’s 447 official neighborhoods [i.e. ≥29.7 %; 29]. This indicator was not available for four of our 40 neighborhoods. The average proportion of long-term unemployment of surrounding neighborhoods was used as an approximation for the missing data.

#### Self-report information

Self-report information was used to compare participants and NP+. Self-report information for participants was based on the CAPI, except for information on quality of life which was assessed by self-administered questionnaire.

Living arrangements was dichotomized into *married and living together* (yes/no).

*School education* was categorized as <10, 10, or >10 years.

Long-standing or *chronic disease* was assessed with one question from the Minimum European Health Module [30]. “Do you have any chronic illness or some long-standing health problem, e.g., diabetes or a heart disease?” (yes/no). For participants, chronic diseases were additionally defined as “long-standing illnesses that need continuous treatment and monitoring”.

Health-related *quality of life* was assessed by the EQ-5D-3 L of the EuroQol Group [31, 32]. Five dimensions with a three-answer format determine problems with mobility, self-care, the performing of usual activities, the extent of pain/discomfort and anxiety/depression. A total score was calculated (range: 0–100). In addition, answer categories in all five dimensions were dichotomized (yes/no): at least some *mobility problems*, at least some *self-care problems,* at least some *problems performing usual activities,* moderate/extreme *pain/discomfort* and moderate/extreme *anxiety/depression*.

*Polypharmacy* was assessed by one or two self-developed questions for NP+ and participants, respectively. NP+ were asked “How many different prescribed medications do you take?” (none; 1–3; 4–6; >6). Participants were asked “Do you currently (in the last 7 days) take prescribed medications?” If response was positive, participants were asked how many medications. This information was combined to consider taking ≥4 prescribed medications as an indicator of *polypharmacy* (yes/no).

*Need for assistance* was assessed by one question modified from the German Ageing Survey [33]. “At the moment, are you dependent on others to cope with everyday life, e.g., for personal hygiene, cleaning, personal and financial organization, because of a chronic illness or some long-standing health problem?” For participants, the current need for support was further defined as “in the last seven days”.

Based on Minder et al. [20], detailed reasons were summarized into a *main reason for non-participation* in the following hierarchical order: being too healthy, being too ill, other reasons and no interest. For example, an individual who reported ill health and limited knowledge of German was categorized as being too ill. In contrast, an individual was only categorized as having no interest if no health-related or other reasons were stated.

### Data analyses

Statistical analyses were performed using IBM SPSS Statistics 20 [34] and Stata/SE 12.1 [35]. First, descriptive statistics and 95 % confidence intervals (95 %-CIs) using Wilson’s method were calculated [36]. Second, two multivariable multinomial regression analyses were applied to determine all group differences for the register-based information using participants (analysis 1) or NP+ (analysis 2) as the reference group. Because the sample clustered in 40 different neighborhoods and aggregated data at this level were included in the regression models, adjustments in calculating the standard errors and confidence intervals were required and survey procedures with Taylor linearization and neighborhoods as primary sampling unit were applied in Stata/SE 12.1 [37]. Third, bivariable and multivariable logistic regression models were applied to determine group differences between P and NP+ for each self-reported variable. Subjects with missing values were not included in the logistic regression models. Relative risk ratios (RRRs) plus 95 %-CIs, and odds ratios (ORs) plus 95 %-CIs are presented for multinomial and logistic regression models, respectively. *P*-values at the 5 % level and lower were considered significant.

## Results

### Sample characteristics

**Fig. 1:**
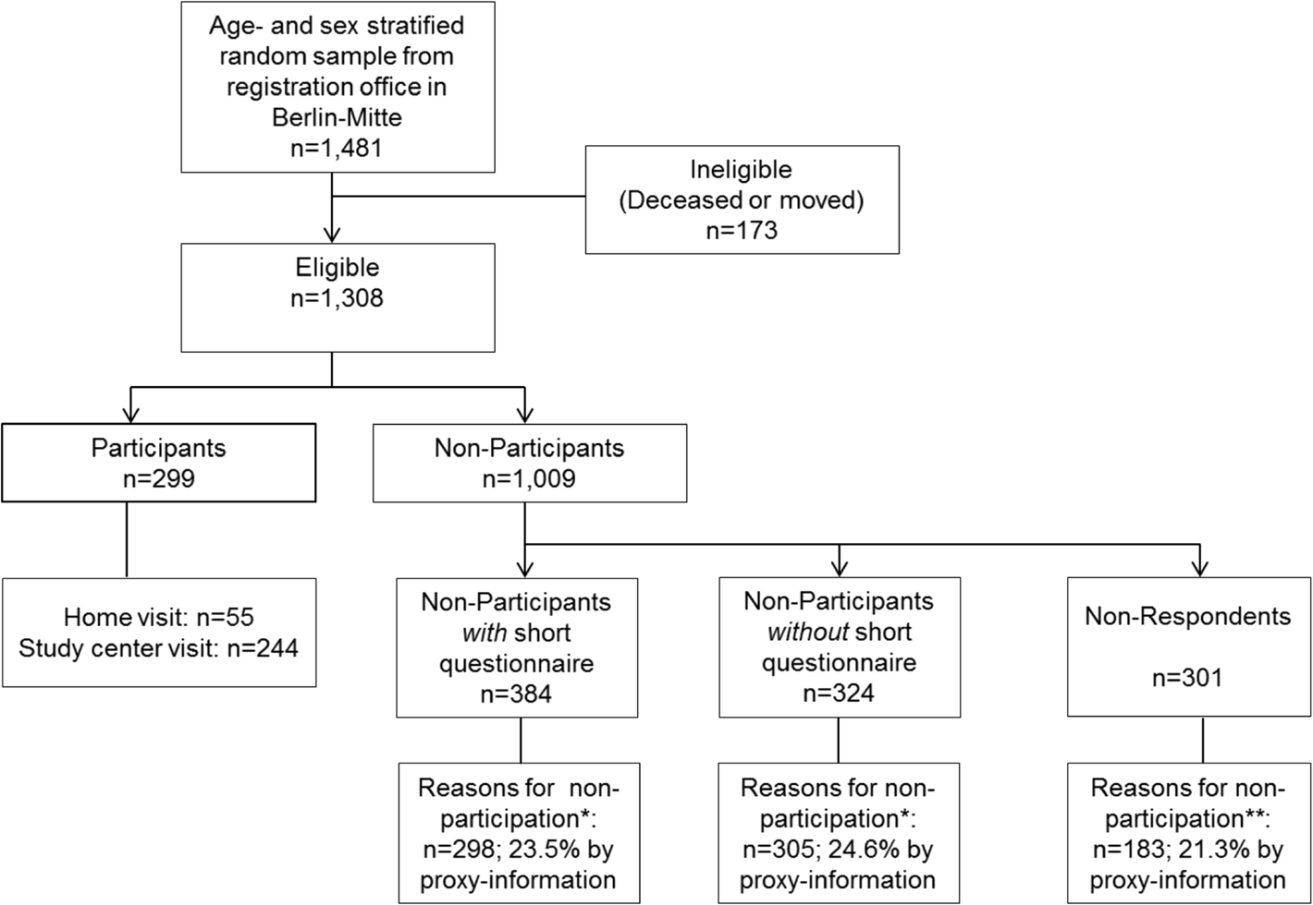
Flow Chart for Baseline Participation. Legend of Fig. 1. * Reasons for non-participation stated during recruitment period in 2009. ** In 2010, non-respondents were given an opportunity to report retrospectively a reason for non-participation

In total, 173 of 1481 individuals were ineligible for the study (Figure 1). Of the remaining 1308 eligible individuals, 299 (22.9 %) took part in the complete study protocol at baseline. Overall, 55 of the 299 assessments were conducted at the participants’ homes. Of the 1009 non-participants, 384 were NP+, 324 were NP-, and 301 were NR. Participation was declined by proxy information in 77 of the NP+ and 75 of the NP-. Sample characteristics for the total sample and different subgroups are shown in Table 1. The mean age of the total sample was 77.2 years (SD = 7.6); 49.4 % were women, 11.5 % had non-German citizenship, 4.1 % lived in residential care and 35.4 % lived in a deprived neighborhood.

**Table 1:** Sample Characteristics for the Total Sample (*n* = 1481) and Different Subgroups

	Total sample							
	Total	Ineligible	Eligible					
			Total	Participants	Non-participants			
					Total	NP+	NP-	NR
	*n* = 1481	*n* = 173	*n* = 1,308	*n* = 299	*n* = 1009	*n* = 384	*n* = 324	*n* = 301
Women - % (95 %-CI)	49.4 (46.8, 51.9)	44.5 (37.3, 52.0)	50.0 (47.3, 52.7)	45.8 (40.3, 51.5)	51.2 (48.2, 54.3)	54.7 (49.7, 59.6)	51.9 (46.4, 57.2)	46.2 (40.6, 51.8)
Age in years – M (SD)	77.2 (7.6)	80.0 (8.8)	76.8 (7.4)	75.0 (6.6)	77.4 (7.5)	78.1 (7.3)	77.7 (7.6)	76.0 (7.5)
Age groups - % (95 %-CI)								
65–74 years	41.9 (39.4, 44.4)	30.1 (23.7, 37.3)	43.4 (40.8, 46.1)	53.8 (48.2, 59.4)	40.3 (37.4, 43.4)	33.6 (29.1, 38.5)	39.5 (34.3, 44.9)	49.8 (44.2, 55.4)
75–84 years	40.6 (38.1, 43.1)	39.3 (32.3, 46.7)	40.7 (38.1, 43.4)	35.8 (30.6, 41.4)	42.2 (39.2, 45.3)	48.4 (43.5, 53.4)	40.7 (35.5, 46.2)	35.9 (30.7, 41.4)
≥ 85 years	17.6 (15.7, 19.6)	30.6 (24.2, 37.9)	15.8 (13.9, 17.9)	10.4 (7.4, 14.3)	17.4 (15.2, 19.9)	18.0 (14.5, 22.1)	19.8 (15.8, 24.4)	14.3 (10.8, 18.7)
Non-German citizenship - % (95 %-CI)	11.5 (10.0, 13.3)	38.7 (31.8, 46.2)	8.0 (6.6, 9.5)	2.7 (1.4, 5.2)	9.5 (7.9, 11.5)	3.4 (2.0, 5.7)	7.4 (5.0, 10.8)	19.6 (15.5, 24.5)
Residential care - % (95 %-CI)	4.1 (3.2, 5.3)	9.2 (5.8, 14.5)	3.4 (2.6, 4.6)	0.7 (0.2, 2.4)	4.3 (3.2, 5.7)	6.0 (4.0, 8.8)	4.0 (2.4, 6.7)	2.3 (1.1, 4.7)
Deprived neighborhood - % (95 %-CI)	35.4 (33.0, 37.9)	43.4 (36.2, 50.8)	34.3 (31.8, 36.9)	28.1 (23.3, 33.4)	36.2 (33.3, 39.2)	35.7 (31.0, 40.6)	37.0 (32.0, 42.4)	35.9 (30.7, 41.4)

Abbreviations: 95 %-CI = 95 % confidence interval; M = mean; NP + = non-participants with the short questionnaire, i.e., individuals who declined study participation but completed the short questionnaire; NP- = non-participants without the short questionnaire, i.e., individuals who declined study participation and completing the short questionnaire; NR = non-respondents, i.e., individuals who could not be reached during the recruitment period and who did not actively decline study participation; SD = standard deviation

### Differences between participants and the three subgroups of non-participants at baseline

Multinomial regression with participants as the reference group revealed that NP+ were more often older, female and lived more often in residential care (Table 2). NP- were older, more often had non-German citizenship and lived more often in deprived neighborhoods than participants. NR had non-German citizenship more often than participants. All other comparisons between participants and the three subgroups of non-participants were not significant.

**Table 2:** Group Differences Based on Multivariable Multinomial Logistic Regressions at Baseline (*n* = 1308)^a^

	Analysis 1			Analysis 2	
	P = reference group			NP + = reference group	
	NP+	NP-	NR	NP-	NR
	RRR (95 %-CI)	RRR (95 %-CI)	RRR (95 %-CI)	RRR (95 %-CI)	RRR (95 %-CI)
Sex					
Men	Ref.	Ref.	Ref.	Ref.	Ref.
Women	1.41 (1.04, 1.93)	1.29 (0.94, 1.77)	1.10 (0.81, 1.49)	0.91 (0.67, 1.24)	0.78 (0.58, 1.05)
Age groups in years					
65–74	Ref.	Ref.	Ref.	Ref.	Ref.
75–84	2.16 (1.49, 3.13)	1.59 (1.05, 2.40)	1.20 (0.84, 1.72)	0.74 (0.50, 1.09)	0.56 (0.41, 0.76)
≥ 85	2.49 (1.56, 3.97)	2.55 (1.43, 4.56)	1.67 (0.90, 3.11)	1.03 (0.66, 1.58)	0.67 (0.42, 1.09)
Citizenship					
German	Ref.	Ref.	Ref.	Ref.	Ref.
Non-German	1.58 (0.61, 4.14)	3.39 (1.39, 8.24)	9.41 (4.07, 21.75)	2.14 (1.10, 4.19)	5.95 (3.03, 11.69)
Residential care					
No	Ref.	Ref.	Ref.	Ref.	Ref.
Yes	7.99 (1.58, 40.35)	5.11 (0.98, 25.56)	3.72 (0.73, 18.95)	0.64 (0.39, 1.05)	0.47 (0.24, 0.92)
Deprived neighborhood					
No	Ref.	Ref.	Ref.	Ref.	Ref.
Yes	1.37 (0.95, 1.97)	1.46 (1.08, 1.97)	1.33 (0.87, 2.04)	1.07 (0.77, 1.48)	0.97 (0.67, 1.43)

Abbreviations: 95 %-CI = 95 % confidence interval; NP + = non-participants with the short questionnaire, i.e., individuals who declined study participation but completed the short questionnaire; NP- = non-participants without the short questionnaire, i.e., individuals who declined study participation and completing the short questionnaire; NR = non-respondents, i.e., individuals who could not be reached during the recruitment period and who did not actively decline study participation; P = participants; Ref. = reference category; RRR = relative risk ratio.

^a^ Adjusted for clustering within neighborhoods

To identify differences among the three non-participants subgroups, the same analysis was repeated with NP+ as the reference group. NP- had non-German citizenship more often than did NP+. NR were younger, had non-German citizenship more often and were less often in residential care than NP+. All other comparisons between the three subgroups of non-participants were not significant.

### Differences between participants and non-participants *with* the short questionnaire at baseline

Unadjusted bivariate logistic regressions to predict baseline non-participation were conducted to analyze group differences (participants vs. NP+) in living arrangements, education, and selected health indicators (Table 3). There was no significant group difference regarding being married and living together. Compared with participants, NP+ had significantly lower school education, had ≥ 1 chronic disease less often, reported problems within all five EQ-5D dimensions more frequently as well as lower mean overall EQ-5D quality of life scores. In addition, NP+ reported polypharmacy less often and were more often in need of assistance than participants.

**Table 3:** Association between Participation Status and Key Health Characteristics comparing Participants (0) and Non-participants *with* Short Questionnaire (1) at Baseline (Valid Cases: *n* = 635)

		P (*n* = 299)	NP+ (*n* = 384)	Modell 1^b^			Modell 2^c^		
*Self-report information*	*n*	%/M (SD)^a^	%/M (SD)^a^	OR	95 %-CI	*p*	OR	95 %-CI	*p*
Married and living together	682	53.0	46.9	0.78	0.58, 1.06	0.521	1.05	0.75, 1.46	0.792
School education years	671								
< 10		39.9	56.5	Ref.			Ref.		
10		26.7	23.5	0.62	0.42, 0.90	0.013	0.61	0.41, 0.90	0.013
>10		33.4	20.0	0.42	0.29, 0.61	<0.001	0.51	0.35, 0.76	0.001
≥ 1 chronic disease	679	80.5	64.3	0.44	0.31, 0.62	<0.001	0.37	0.25, 0.54	<0.001
Quality of life (Total score)	653	78.7 (16.9)	67.1 (23.3)	0.97	0.96, 0.98	<0.001	0.98	0.97, 0.99	<0.001
Mobility problems	679	28.6	51.8	2.68	1.95, 3.70	<0.001	2.07	1.47, 2.91	<0.001
Self-care problems	677	10.1	29.2	3.67	2.37, 5.69	<0.001	2.48	1.55, 3.96	<0.001
Problems performing usual activities	677	23.9	44.7	2.58	1.84, 3.60	<0.001	1.83	1.27, 2.62	0.001
Pain/discomfort	662	59.4	69.7	1.57	1.14, 2.17	0.006	1.36	0.98, 1.91	0.069
Anxiety/depression	659	18.7	34.9	2.34	1.62, 3.38	<0.001	2.03	1.39, 2.97	<0.001
Poly-pharmacy	678	55.7	47.9	0.73	0.54, 0.99	0.044	0.59	0.42, 0.81	0.001
Need for assistance	678	16.7	32.5	2.39	1.65, 3.47	<0.001	1.49	0.99, 2.26	0.056

Abbreviations: 95 %-CI = 95 % confidence interval; M = mean; NP + = non-participants with the short questionnaire, i.e., individuals who declined study participation but completed the short questionnaire; OR = odds ratio; P = participants; Ref. = reference group; SD = standard deviation.

^a^ % for categorical variables. M (SD) for continuous variables

^b^ Unadjusted bivariate logistic regressions

^c^ Modell 1 + adjusted for age, sex, residential care

After adjustment for age in years, sex and living in residential care, group differences remained significant. There were two exceptions: need for assistance and the EQ-5D dimension pain/ discomfort were no longer significantly different between NP+ and participants.

### Reasons for non-participation at baseline

During the recruitment period in 2009, for 603 of the 1009 non-participants at least one reason for non-participation at baseline could be obtained (see Fig. 1). On average, 1.4 reasons per person (SD = 0.7; Median = 1; 75^th^ percentile = 2; Maximum = 5) were reported in 2009; 394 of these 603 non-participants (65.3 %) specified one reason, and 458 target persons reported the reasons for non-participation themselves (76.0 %).

During the retrospective period in 2010, it was possible to identify reasons for non-participation at baseline for 183 of the 301 NR. On average, 1.4 reasons per person (SD = 0.7; Median = 1; 75^th^ percentile = 2; Maximum = 4) were stated in 2010; 125 of the 183 NR specified one reason (68.3 %) and 141 target persons reported the reasons for non-participation themselves (77.0 %). Among 118 persons who could again not be reached, 44.1 % were not reached at their home address despite multiple personal and postal contact attempts, 26.3 % had moved to an unknown address, 13.6 % had died, 10.2 % were permanently absent according to proxy information and 5.9 % had moved out of the area.

Table 4 presents the reasons for baseline non-participation. In total, a reason was given by 786 of the 1009 non-participants (77.9 %). Based on the rankings from 2009 to 2010, the top five reasons were similar. However, the remaining reasons were ranked differently; a larger variety of reasons was given in 2009 than in 2010.

**Table 4:** Detailed Reasons for Baseline Non-participation Stated in 2009 or 2010^a^ by Non-participants (*n* = 786)

	Rank order total	Rank order 2009	Rank order 2010	Total	NP+	NP-	NR
	(*n* = 786)	(*n* = 603)	(*n* = 183)	(*n* = 786)	(*n* = 298)	(*n* = 305)	(*n* = 183)
*Detailed reasons (multiple reasons permitted) - %*							
Refusal to participate in scientific studies on principle	1	1	2	42.1	37.2	53.4	31.1
Too ill	2	2	2	31.4	38.3	24.9	31.1
No interest in this study	3	3	1	25.8	16.1	25.9	41.5
No time^b^	4	4	4	12.5	18.1	7.5	11.5
Limited knowledge of German^b^	5	5	5	4.6	0.7	5.9	8.7
Dementia	6	11	6	3.1	3.4	1.0	6.0
Miscellaneous^b^	7	7	9	2.7	2.3	3.9	1.1
Too old^b^	7	7	9	2.7	4.0	2.3	1.1
Participation too strenuous^b^	9	5	-	2.5	4.7	2.0	0.0
Family member too ill/ in need of care^b^	10	10	13	1.9	3.7	1.0	0.5
Hospital stay	10	9	-	1.9	2.3	2.6	0.0
Hearing/visual impairment	12	12	9	1.7	2.0	1.6	1.1
Personal reasons (not otherwise specified)^b^	13	13	-	1.1	1.7	1.3	0.0
Having sufficient medical care^b^	13	13	-	1.1	2.7	0.3	0.0
Having no personal benefit^b^	15	15	-	1.0	0.7	2.0	0.0
Too healthy	16	16	-	0.9	2.0	0.3	0.0
Death of a family member^b^	16	16	-	0.9	1.3	1.0	0.0
Speech disorder	16	19	9	0.9	1.0	0.7	1.1
Being in residential care in 2009	16	25	7	0.9	0.0	0.3	3.3
Too busy^b^	20	18	-	0.8	1.3	0.7	0.0
Being absent during recruitment period^b^	21	-	8	0.6	0.0	0.0	2.7
Participating in another scientific study^b^	22	20	13	0.5	0.3	0.7	0.5
Being in rehabilitation	23	20	-	0.4	0.7	0.3	0.0
Being unmotivated^b^	23	20	-	0.4	0.7	0.3	0.0
Bad experiences with scientific studies^b^	23	20	-	0.4	0.0	1.0	0.0
Having concerns about the study’s risks^b^	26	24	-	0.3	0.3	0.3	0.0

Abbreviations: NP + = non-participants with short questionnaire, i.e., individuals who declined study participation but completed the short questionnaire; NP- = non-participants without the short questionnaire, i.e., individuals who declined study participation and completing the short questionnaire; NR = non-respondents, i.e., individuals who could not be reached during the recruitment period and who did not actively decline study participation

^a^Reasons for non-participation stated during the 2009 recruitment period. In 2010, non-respondents were given an opportunity to report retrospectively a reason of non-participation

^b^These reasons were summarized as “other” if there was no indication of being too healthy or ill

In total, the most frequent (>2.0 %) reasons were ‘refusal to participate in scientific studies on principle’ (42.1 %), ‘being too ill’ (31.4 %), ‘having no interest in the study’ (25.8 %), ‘having no time’ (12.5 %), ‘limited knowledge of German’ (4.6 %), ‘dementia’ (3.1 %), ‘miscellaneous’ (2.7 %), ‘being too old’ (2.7 %) and anticipating ‘participation as too strenuous’ (2.5 %). Among NP- and NR, ‘refusal to participate in scientific studies on principle’ or ‘no interest in this study’ were the most-often reported reasons, followed by ‘being too ill’. NP+ reported ‘being too ill’ and ‘having no time’ more often than did the two other groups. In contrast, ‘limited knowledge of German’ was reported more often by NP- and NR compared with NP+. ‘Dementia’, ‘being absent during the recruitment period’ or ‘being in residential care’ were more often reasons for non-participation by NR compared with the two other groups.

**Fig. 2:**
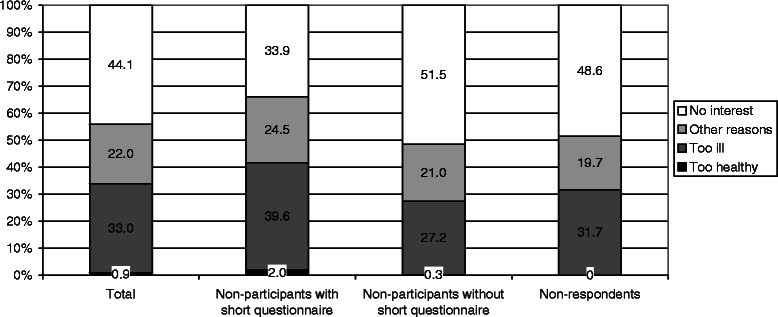
Main Reason for Baseline Non-participation by Non-participation Group (*n* = 786)

Groups differed according to their main reason for baseline non-participation (Fig. 2). NP+ described themselves more often as being too ill, more often had other reasons and were less often not interested compared with the two other groups.

## Discussion

This register-based study of adults 65 years and older aimed to estimate baseline response biases and to highlight the diversity of non-participants. We applied a three-step approach to collect information and differentiated between study participants, non-participants who at least answered a standardized health questionnaire (NP+), non-participants who actively declined to provide any information (NP-), and non-responders (NR). Information on main study characteristics (age, sex, non-German citizenship as an indicator of immigration background, living in a socially deprived area) and nursing home residence was available for the full sample. At the second level, information on education, living arrangements, health status, health-related quality of life, and need for assistance was available for a total of 683/1308 or 52.2 % of the net sample, including study participants and NP+. Finally, detailed information on reasons for non-participation was obtained for a total of (786/1009) or 77.9 % of all non-participants including 183 non-responders for whom this information could be obtained retrospectively.

As expected, the proportion of eligible persons who participated in the interview and examination was small (299/1308). Compared to the net sample, persons with non-German citizenship, persons 85 years and older, those living in deprived neighborhoods, and nursing home residents were underrepresented among study participants. Non-German citizenship was the single most important independent determinant of non-response or non-participation with decline to provide any health information.

In accordance with others [e.g., 20, 26], non-participants were a heterogeneous group. Ill health, limited German language proficiency, dementia frailty and being in the hospital ranked among the top 10 reasons, along with restraints to participate in scientific studies in principle or in this particular study and lack of interest or time.

Compared with participants, NP+ lived more often in residential care and reported lower health-related quality of life; they were also less likely to report at least one chronic disease or polypharmacy. One possible explanation for these seemingly contradictory results may be lower health service utilization among non-participants compared with participants. This could reduce the probability to receive a medical diagnosis or treatment despite functional limitations. Less education, as in our study, could be an underlying determinant and act as a barrier in health care utilization in this subgroup [38]. More research is needed to explain and verify this finding. However, we cannot rule out that measurement and mode effects caused these findings because the wording of the questions on chronic disease and need for support varied slightly between the two groups. In addition, participants took part in a brown bag medication review. They could have been more aware of their medications intake than NP+.

NP- and NR belonged more often to disadvantaged groups (non-German citizenship; living in deprived neighborhoods) compared with participants at baseline. Socially disadvantaged individuals are at higher risk of diseases [e.g., 39]. In longitudinal studies, higher rates of morbidity [40–42] and mortality [4, 20, 26, 40, 42] have been reported for non-participants vs. participants. Subgroup analyses found especially worse outcomes for those non-participants who were too ill and for NR compared with participants [26, 40]. Only eight participants died within the first year of follow-up of our study [see 28]. Unfortunately, it was not possible to conduct a mortality follow-up of the entire study cohort (i.e. the subgroups of non-participants).

Obviously, there are difficulties in recruiting representative baseline samples of the population 65+ years for population register-based health studies. In particular, individuals 85+ years and those from disadvantaged subgroups are missing from the studies. In Germany as in other high-income countries, persons 80+ years are the fasted growing group of the population [43]. Currently, the risk of poverty among older people is relatively low, but socioeconomic projections indicate, that poverty rates among older people might increase in future cohorts [44]. In order to provide valid estimates of morbidity, functional capacities, health risks and health care needs in the population 65+ years, it is necessary to develop specific recruitment strategies tailored to the oldest old and to underrepresented groups; existing efforts do not appear to be sufficient. As a first example, a brief description of our study was offered in several languages to overcome language barriers, but this description was rarely requested. As a second example, only 55 individuals used the home visit offer. Possibly, tailoring recruitment strategies to older persons and including gatekeepers, such as family physicians, home care nurses or social workers could be helpful [19, 45]. However, in health studies aimed at the population 65 years and older at large, register-based sampling strategies may not be effective at all to achieve representative and sufficiently large samples of subgroups that are difficult to reach. Sampling strategies therefore need reconsideration. Multiple sampling frames may be useful to estimate a small set of key health indicators for the older population at large and to collect additional information relevant to specific subgroups also using proxy information [19, 46]. Register-based samples could be complemented by additional samples drawn at places where sufficiently large numbers of individuals can be approached in an atmosphere of trust and care, such as nursing homes, home nursing care networks, family physicians, dentists, adult day care centers for seniors or regular meetings for older persons organized by churches.

To obtain a better understanding of the moderate total participation rate in our study at baseline, the analysis of reasons for non-participation is helpful. Of all non-participants, no interest was the main reason for non-participation, followed by ill health, which is in line with the literature [8, 10, 12, 16]. In contrast to previous studies [20, 26], we found that only a marginal percentage of non-participants described themselves as ‘too healthy’ (*n* = 7). Although a group of ‘too healthy’ individuals might exist across countries, its percentage and influence on bias could vary. The frequency of reasons differed by non-participant subgroup. ‘Refusal to participate in scientific studies on principle’ and ‘no interest’ were more frequently cited among NR and NP-. In contrast, ‘being too ill’ was more often reported by NP+, which is in line with our observation of lower health-related quality of life in this group compared to study participants. The living situations of older individuals are heterogeneous and complex. When planning health studies including older individuals, this circumstance should be considered, and field workers should be provided with appropriate strategies.

The major strength of our study is that we were able to estimate baseline non-response bias and to highlight the diversity of non-participants by applying a three-step approach to collect information. Some limitations apply to our study. First, we obtained a total baseline participation rate of 52 % and a participation rate for the complete study protocol of only 23 %. A number of factors are likely to have contributed to this finding: (a) the application of very few exclusion criteria, e.g., language problems and dementia were not exclusion criteria; (b) the inner-city resident sample, others found worse participation rates in urban vs. rural areas [e.g., 3, 47–49] - especially in inner cities [50]; and (c) the oversampling of the oldest old, who have the lowest participation rates [see also 15]. Second, some of the reasons for non-participation were obtained retrospectively. Akhtar [11] reported that only 30 % indicated the same reason for non-participation one year later. However, individuals in this previous study were interviewed once by a health visitor and once by a physician, which might have added to this effect and which was not the case in our study. Third, the diagnosis of dementia was not verified by medical records; only proxy information was given.

Our analyses on baseline non-participation in a health examination study among individuals 65+ years had some advantages. First, we obtained complete register-based information on demographics for the total sample. Therefore, our subgroup analysis also included a non-responder group. Second, the majority of all non-participants (78 %) provided a reason for non-participation, including even 61 % of NR. Third, only a small number of exclusion criteria were applied. In contrast to others [e.g., 20, 38], residential care, insufficient knowledge of the language, and having a terminal illness or dementia were not exclusion criteria. Fourth, the oldest old were included and even oversampled in the study. Finally, a home visit was offered as a standard procedure.

## Conclusions

Our results add to evidence that findings from register-based health surveys of the population 65+ years are likely to be biased as socially deprived, very old persons and with foreign citizenships are underrepresented. In addition to previous studies, we were able to estimate baseline response bias by applying a three-step approach to collect health-related information. This also permitted highlighting the diversity in health problems and barriers to participation among non-participants. Innovative sampling strategies using multiple sampling frames are needed for health surveys in aging populations to achieve valid estimates of health status, health risks and health care needs for the population 65+ years at large including hard-to-reach population subgroups with specific health care needs.

## Abbreviations

CAPI: computer-assisted personal interview
95 %-CI: 95 % confidence interval
M: mean
NP-: non-participants without a short questionnaire, i.e. individuals who had declined study participation as well as the completion of the short questionnaire
NP+: non-participants with a short questionnaire, i.e. individuals who had declined study participation, but who had filled in the short questionnaire
NR: non-respondents, i.e. individuals who could not be reached during the recruitment period and who did not actively decline study participation
OMAHA: Operationalizing Multimorbidity and Autonomy for Health Services Research in Aging Populations
OR: odds ratio
P: participants
Ref.: reference category
RRR: relative risk ratio
SD: standard deviation
